# Time lapse analysis of tumor response in patients with soft tissue sarcoma treated with trabectedin: A pooled analysis of two phase II clinical trials

**DOI:** 10.1002/cam4.2991

**Published:** 2020-03-27

**Authors:** Makoto Endo, Shunji Takahashi, Nobuhito Araki, Hideshi Sugiura, Takafumi Ueda, Tsukasa Yonemoto, Mitsuru Takahashi, Hideo Morioka, Hiroaki Hiraga, Toru Hiruma, Toshiyuki Kunisada, Akihiko Matsumine, Kazato Goda, Akira Kawai

**Affiliations:** ^1^ Department of Orthopaedic Surgery Kyushu University Fukuoka Japan; ^2^ Department of Medical Oncology Cancer Institute Hospital of Japanese Foundation for Cancer Research Tokyo Japan; ^3^ Department of Orthopaedic Surgery Osaka International Cancer Institute Osaka Japan; ^4^ Department of Physical Therapy Nagoya University Nagoya Japan; ^5^ Department of Orthopaedic Surgery National Hospital Organization Osaka National Hospital Osaka Japan; ^6^ Division of Orthopaedic Surgery Chiba Cancer Center Chiba Japan; ^7^ Division of Orthopaedic Surgery Shizuoka Cancer Center Hospital Nagaizumi Japan; ^8^ Department of Orthopaedic Surgery National Hospital Organization Tokyo Medical Center Tokyo Japan; ^9^ Department of Orthopaedic Surgery Hokkaido Cancer Center Sapporo Japan; ^10^ Department of Musculoskeletal Tumor Surgery Kanagawa Cancer Center Yokohama Japan; ^11^ Department of Orthopaedic Surgery Okayama University Hospital Okayama Japan; ^12^ Department of Orthopaedic Surgery Fukui University Fukui Japan; ^13^ Department of Medical affairs Taiho Pharmaceutical Co. Ltd Tokyo Japan; ^14^ Department of Musculoskeletal Oncology National Cancer Center Hospital Tokyo Japan

**Keywords:** chemotherapy, clinical trial, soft tissue sarcoma

## Abstract

The time course of the response to each drug is important to avoid inappropriate termination of treatment by misjudging tumor progression; however, little is known about soft tissue sarcoma (STS) regarding this matter. This study aimed to perform a time‐lapse analysis of tumor response in patients with STS treated with trabectedin from 2 phase II clinical trials.

We examined 66 patients with translocation‐related sarcoma registered in 2 Japanese phase II clinical trials. All patients previously received standard therapy before the administration of trabectedin at 1.2 mg/m^2^ every 3 weeks. Imaging evaluation was performed according to the study protocol. The sum of the maximum diameters of the target lesions was calculated and analyzed over time.

Among the 66 patients, 9 (13.6%) showed partial response (PR) to trabectedin. Histological diagnoses of these 9 responders comprised 6 myxoid liposarcoma, 2 synovial sarcoma, and a mesenchymal chondrosarcoma. The median period from treatment initiation to the first PR was 123 (range, 34‐328) days. The pattern of tumor response to trabectedin showed an increasing tendency in size in the initial stage, usually followed by a size decrease with repeated administration.

STS response to trabectedin was characterized as delayed and potentially persistent. Clinicians treating STS with trabectedin should know the features of the response pattern to avoid interrupting the treatment before maximal efficacy is achieved.

## Graphical abstract

Among 66 patients with translocation‐related sarcoma, 9 showed partial response to trabectedin, and 5 of the 9 responders (56%) fulfilled the criteria for partial response later than 100 days from treatment initiation. Tumor response to trabectedin was characterized as delayed and stabilized by the detailed time‐lapse analysis of tumor size.

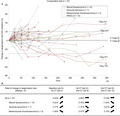

## 1 INTRODUCTION

Treatment goals for patients with metastatic sarcoma include suppressing tumor progression as long as possible to delay or reduce concomitant symptoms and increasing survival while maintaining the quality of life. When there is no risk of cumulative toxicity, treatment can be continued as long as antitumor activity is observed and adverse events remain controlled; however, if tumor progresses, drug therapy is usually terminated or changed. Immune checkpoint inhibitors have a unique response pattern known as pseudo‐ or hyperprogression, which is characterized by an increase in size during the initial stage of treatment followed by delayed tumor shrinkage. Therefore, tumor response to immune checkpoint inhibitors cannot be appropriately evaluated by the conventional Response Evaluation Criteria in Solid Tumors (RECIST) alone.^1^

In real‐life practice, a limited number of drugs are available for treating rare cancers, including soft tissue sarcoma (STS). Therefore, it is important to choose the appropriate drug for each patient and to use it properly as long as the drug maintains its efficacy. Clinicians should know the time course of STS response to each drug to avoid inappropriate termination of treatment by misjudging tumor progression; however, little is known about that of STS.

Trabectedin is a cytotoxic anticancer agent that acts by binding to DNA and disrupting DNA repair mechanisms.^2^ In several clinical trials involving patients with metastatic and/or recurrent STS, trabectedin controlled the disease activity favorably compared with best supportive care (BSC) or dacarbazine.^3^, ^4^ Since then, trabectedin has been used, mainly as a second‐line or later treatment, for advanced STS cases. In the clinical settings, trabectedin often starts reducing the tumor size after several cycles, with the tumor remaining unchanged or even increasing in size slightly during the early stage of therapy. However, no detailed time‐lapse analyses of tumor response to trabectedin has been conducted. This study aimed to analyze the pattern of tumor response in patients with STS treated with trabectedin using data from prospective phase II clinical trials in Japan.

## 2 MATERIALS AND METHODS

### 2.1 Trial information

**Figure 1:**
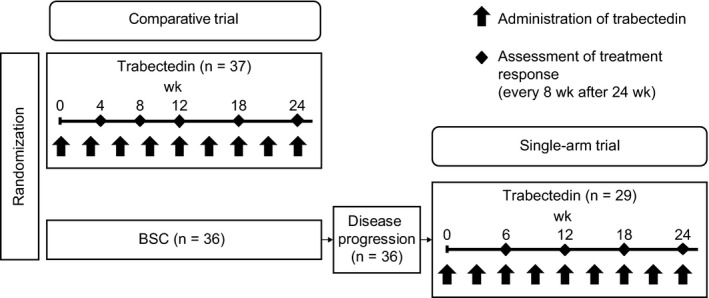
Flow diagram. BSC, best supportive care

This study included patients treated with trabectedin who were registered in 2 phase II clinical trials conducted in Japan (comparative trial, no. JapicCTI‐121850; single‐arm trial, no. JapicCTI‐121853, Figure 1).^4^, ^5^ The trials were approved at each participating medical facility and performed according to the Declaration of Helsinki and the Japanese Good Clinical Practice guidelines. Written informed consent was obtained from all subjects registered in the trials.

### 2.2 Patients

Patients with histologically proven translocation‐related sarcoma and who previously received standard therapy were included.^6^ In all patients, trabectedin was administered at a dosage of 1.2 mg/m^2^ every 3 weeks according to the results of the preceding phase I study conducted in Japan.^7^ Trabectedin treatment was delayed until the following criteria were met; neutrophil count more than 1500 cells per μL, platelet count more than 10  × 10^4^ cells per μL, blood albumin more than 2.5 g/dL, total bilirubin less than 1.5 mg/dL, aspartate aminotransferase (AST), alanine aminotransferase (ALT), and creatine phosphokinase (CPK) less than 2.5 times of upper limit of normal, and creatinine clearance more than 30 mL per minutes. Dose reduction of trabectedin was considered in the case of grade 3 or 4 adverse events, including thrombocytopenia of less than 25  × 10^3^ cells per μL, neutropenia less than 500 cells per μL lasting for more than 5 days or associated with fever or infection, and increased concentrations of AST and ALT more than 2.5 times of upper limit of normal that had not recovered by day 21. The protocol treatments were continued until disease progression, intolerable toxicity, physician’s decision, or patient’s withdrawal of consent. Disease progression was defined as clinical meaningful tumor increase, and clinician could continue trabectedin treatment after RECIST PD. Subjects initially assigned to the BSC group of the comparative trial were later registered in the single‐arm trial if they intended to receive trabectedin after being rated as having progressive disease (PD) with BSC.

### 2.3 Analysis

Diagnostic imaging was performed according to time points detailed in Figure 1. Lesions were evaluated according to RECIST version 1.1 by the Independent Evaluation Committee. Baseline imaging evaluation was performed within 14 days before registration, and the first trabectedin dose was administered within 8 days after registration. Treatment was continued until a severe adverse event or clinically evident disease progression activity occurred, which was defined as “progression evident on diagnostic images” or “signs of clinically evident progression such as symptom aggravation.” After treatment, diagnostic imaging was repeated until disease progression met the RECIST definition. We defined the responder as the patients who experienced at least a 30% decrease in the sum of diameters of target lesions compared to baseline.

## 3 RESULTS

**Figure 2:**
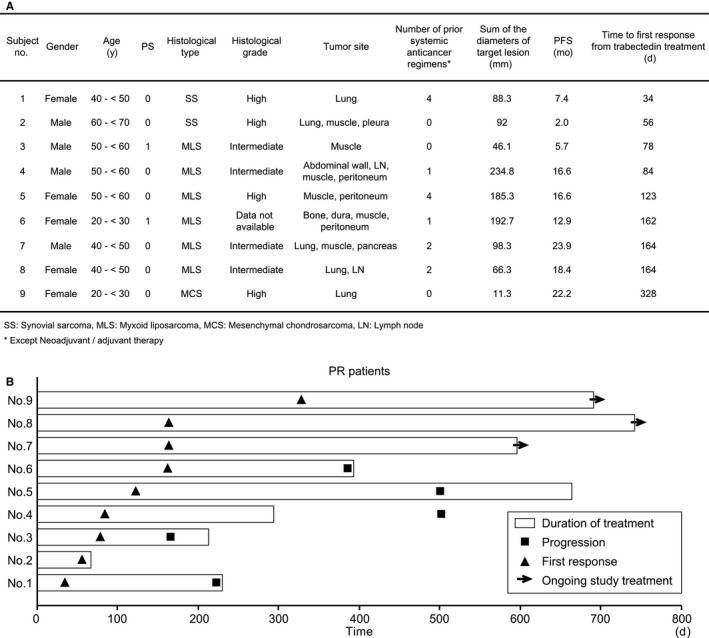
A, Detailed characteristics of patients with PR. PR, partial response; SS, synovial sarcoma; MLS, myxoid liposarcoma; MCS, mesenchymal chondrosarcoma; LN, lymph node. ^†^Except Neoadjuvant/ adjuvant therapy. B., Swimmer plot of 9 PR patients treated with trabectedin. Horizontal line, dosing period; ■, progression; ▲, first response

Overall, 66 patients received trabectedin treatment in the 2 phase II trials that enrolled candidates between July 11, 2012 and January 30, 2014. Patients’ characteristics are summarized in Table 1. Major histological diagnoses which were confirmed by central pathology review board included myxoid liposarcoma (n = 22), synovial sarcoma (n = 17), and mesenchymal chondrosarcoma (n = 6). Among 66 patients in total, 9 showed partial response (PR) to trabectedin; their detailed characteristics and the response timeline are presented in Figure 2A,B. The median time to response from treatment initiation was 123 (range: 34‐328) days.

**Table 1:** Characteristics of patients

	Pooled data (n = 66)	Comparative trial (n = 37)	Single‐arm trial (n = 29)
Sex			
Male, n (%)	38 (57.6)	21 (56.8)	17 (58.6)
Female, n (%)	28 (42.4)	16 (43.2)	12 (41.4)
Age (y)			
Median (range)	38.0 [21, 77]	39.0 [21, 77]	38.0 [25, 60]
PS			
0, n (%)	38 (57.6)	22 (59.5)	16 (55.2)
1, n (%)	28 (42.4)	15 (40.5)	13 (44.8)
Histological type by central pathology review			
Myxoid liposarcoma, n (%)	22 (33.3)	14 (37.8)	8 (27.6)
Synovial sarcoma, n (%)	17 (25.8)	7 (18.9)	10 (34.5)
Mesenchymal chondrosarcoma, n (%)	6 (9.1)	3 (8.1)	3 (10.3)
Extraskeletal Ewing sarcoma, n (%)	5 (7.6)	3 (8.1)	2 (6.9)
Alveolar rhabdomyosarcoma, n (%)	5 (7.6)	2 (5.4)	3 (10.3)
Alveolar soft part sarcoma, n (%)	4 (6.1)	3 (8.1)	1 (3.4)
Extraskeletal myxoid chondrosarcoma, n (%)	2 (3.0)	2 (5.4)	0 (0.0)
Clear cell sarcoma, n (%)	2 (3.0)	1 (2.7)	1 (3.4)
Dermatofibrosarcoma protuberans, n (%)	1 (1.5)	1 (2.7)	0 (0.0)
Angiomatoid fibrous histiocytoma, n (%)	1 (1.5)	1 (2.7)	0 (0.0)
Desmoplastic small round cell tumor, n (%)	1 (1.5)	0 (0.0)	1 (3.4)
Histological grade			
High, n (%)	41 (62.1)	23 (62.2)	18 (62.1)
Intermediate, n (%)	16 (24.2)	8 (21.6)	8 (27.6)
Low, n (%)	2 (3.0)	2 (5.4)	0 (0.0)
Not assessed or unknown, n (%)	7 (10.6)	4 (10.8)	3 (10.3)
Primary lesion			
Lower limbs, n (%)	36 (54.5)	21 (56.8)	15 (51.7)
Abdomen/pelvis, n (%)	7 (10.6)	3 (8.1)	4 (13.8)
Face, n (%)	5 (7.6)	1 (2.7)	4 (13.8)
Intrathoracic, n (%)	3 (4.5)	3 (8.1)	0 (0.0)
Neck, n (%)	3 (4.5)	2 (5.4)	1 (3.4)
Retroperitoneum, n (%)	3 (4.5)	1 (2.7)	2 (6.9)
Other, n (%)	9 (13.6)	6 (16.2)	3 (10.3)
Site by independent radiologic image assessment*			
Lung, n (%)	43 (65.2)	25 (67.6)	18 (62.1)
Peritonea, n (%)	22 (33.3)	12 (32.4)	10 (34.5)
Lymph node, n (%)	20 (30.3)	11 (29.7)	9 (31.0)
Pleura, n (%)	19 (28.8)	11 (29.7)	8 (27.6)
Muscle, n (%)	16 (24.2)	9 (24.3)	7 (24.1)
Bone, n (%)	16 (24.2)	11 (29.7)	5 (17.2)
Other, n (%)	15 (22.7)	10 (27.0)	5 (17.2)
Sum of the diameters of target lesions by independent radiologic image assessment (mm)			
Median (range)	97.65 [10.0, 443.9]	91.70 [10.0, 443.9]	134.20 [25.7, 422.7]
Number of prior regimens of systemic anticancer agents			
Median (range)	1.0 [0,4]	1.0 [0,3]	2.0 [0,4]

Abbreviation: PS, Performance Status.

* Multiple answers allowed.

**Figure 3:**
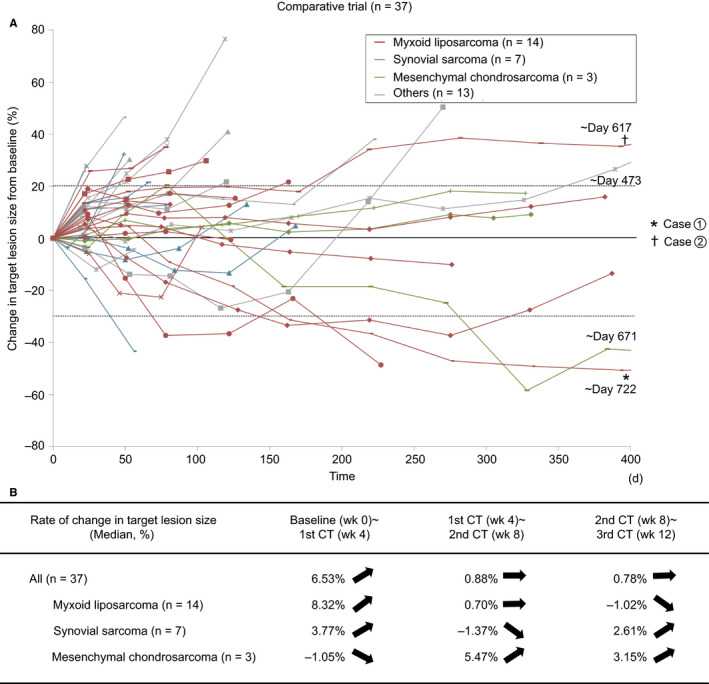
A, Time lapse analysis of tumor size in the comparative trial (n = 37). Day 0 is the day of first imaging measurement before trabectedin treatment. ^†^Case 1 and ^‡^case 2 were presented in Figure 5. B, The rate of change in tumor size between imaging evaluations in patients who completed imaging evaluation at each interval

The time course of changes in tumor diameter in the comparative trial is shown in Figure 3A. The median period from baseline imaging evaluation to trabectedin treatment initiation was 11 days. Figure 3B shows the rate of change in tumor size between the imaging evaluations specified by the study protocol. The pattern of tumor diameter showed an increasing tendency in the initial stage, usually followed by a decrease. The same trend was also observed in the single‐arm study as shown in Figure S1.

**Figure 4:**
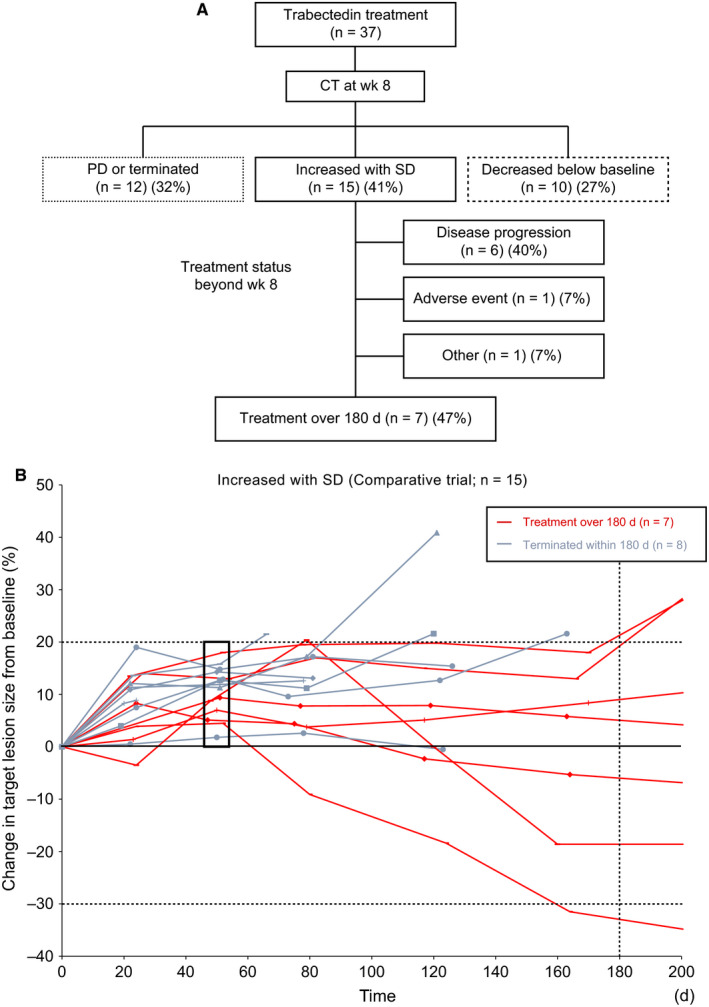
A, Classification based on the change in tumor size on CT at week 8 (n = 37). For the patients with tumor size “increased within SD (stable disease)”, the treatment status beyond week 8 is also shown. B, Time lapse analysis of tumor size limited to the patients with tumor size “increasing within SD” at week 8 (n = 15)

**Figure 5:**
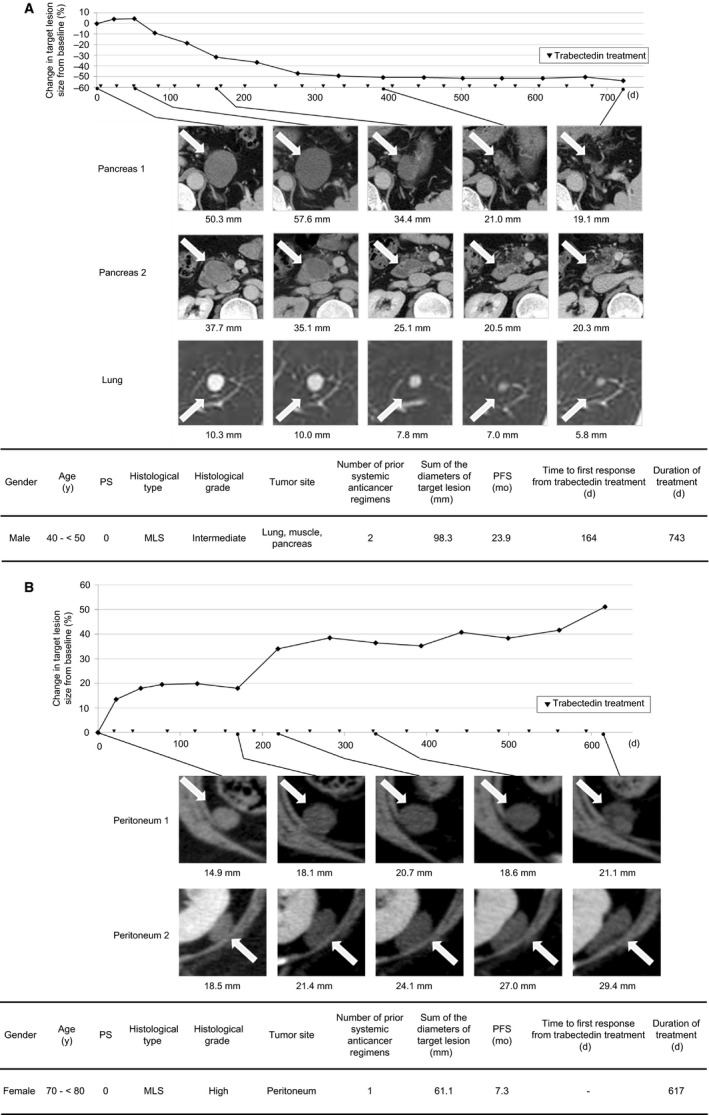
Cases showing delayed response to trabectedin. ▼, trabectedin dose. A, Case 1: male aged 40 y after systemic therapy with doxorubicin, ifosfamide, and cyclophosphamide. After the evaluation of the clinical trial outcome, trabectedin treatment was continued as a form of ordinary clinical care. B, Case 2: female aged 70 y after systemic therapy with doxorubicin

Data of patients in the comparative trial were analyzed to investigate treatment continuity by the initial response category; PD or terminated, increased within SD or decreased below baseline. The results are present in Figure 4A,B. In the comparative trial, computed tomography (CT) was performed every 4 weeks starting from the baseline evaluation. In this study, week 8 was determined to be the timing for the initial assessment of response because week 4 was considered too short to evaluate the response correctly. Among 37 patients who received trabectedin, 15 (41%) had tumors with an increasing tendency at week 8 (growth rate: 0‐20%). Interestingly, seven (47%) of those 15 patients could continue trabectedin for more than 180 days. Among the 66 patients, including those of the single‐arm trial, 23 could continue treatment for more than 180 days. Continuity of trabectedin treatment seemed independent on the initial treatment effectiveness. Diagnostic images of 2 representative cases are shown in Figure 5.

The safety of long‐term trabectedin use is shown in Table 2, which lists adverse events that occurred in at least 15% of patients during the period within 3, 6, to 12, or after 12 months. Besides it, rhabdomyolysis and QT segment prolongation are listed as notable adverse events. In terms of bone marrow suppression, liver dysfunction, cardiac dysfunction, and rhabdomyolysis, there was no evident increase in the incidence with long‐term use of trabectedin. We also investigated treatment cycle delays and dose reductions that occurred during the period within 3, 6, to 12, or after 12 months, as summarized in Table 3. The percentage of patients with cycle delay was 73.9% in total and was 72.5%, 88.0%, and 70.0% in the period of <3 months, 6‐12 months, and >12 months respectively. There was no upward trend in frequency of cycle delay and dose reduction with long‐term administration.

**Table 2:** Frequent or notable adverse events in two clinical trials

	<3 mo	6‐12 mo	>12 mo	All
	(N = 69)	(N = 25)	(N = 10)	(N = 69)
TEAEs, n (%)	69 (100.0)	25 (100.0)	10 (100.0)	69 (100.0)
Blood and lymphatic system disorders				
Anemia	14 (20.3)	5 (20.0)	4 (40.0)	20 (29.0)
Febrile neutropenia	8 (11.6)	2 (8.0)	1 (10.0)	11 (15.9)
Gastrointestinal disorders				
Constipation	37 (53.6)	10 (40.0)	6 (60.0)	42 (60.9)
Diarrhea	10 (14.5)	3 (12.0)	2 (20.0)	13 (18.8)
Nausea	62 (89.9)	15 (60.0)	10 (100.0)	63 (91.3)
Stomatitis	6 (8.7)	4 (16.0)	2 (20.0)	10 (14.5)
Vomiting	29 (42.0)	5 (20.0)	1 (10.0)	31 (44.9)
General disorders and administration site conditions				
Abdominal pain	0 (0.0)	1 (4.0)	2 (20.0)	3 (4.3)
Abdominal pain upper	1 (1.4)	2 (8.0)	2 (20.0)	5 (7.2)
Fatigue	12 (17.4)	2 (8.0)	2 (20.0)	12 (17.4)
Malaise	35 (50.7)	13 (52.0)	5 (50.0)	40 (58.0)
Edema peripheral	4 (5.8)	3 (12.0)	3 (30.0)	10 (14.5)
Pyrexia	12 (17.4)	3 (12.0)	2 (20.0)	19 (27.5)
Infections and infestations				
Pneumonia	0 (0.0)	0 (0.0)	1 (10.0)	1 (1.4)
Upper respiratory tract infection	2 (2.9)	3 (12.0)	2 (20.0)	8 (11.6)
Investigations				
Alanine aminotransferase increased	45 (65.2)	6 (24.0)	3 (30.0)	47 (68.1)
Aspartate aminotransferase increased	36 (52.2)	4 (16.0)	1 (10.0)	39 (56.5)
Blood bilirubin increased	6 (8.7)	0 (0.0)	2 (20.0)	9 (13.0)
Blood creatine phosphokinase increased	6 (8.7)	5 (20.0)	4 (40.0)	11 (15.9)
Gamma‐glutamyltransferase increased	22 (31.9)	9 (36.0)	2 (20.0)	24 (34.8)
Lymphocyte count decreased	12 (17.4)	2 (8.0)	0 (0.0)	15 (21.7)
Neutrophil count decreased	60 (87.0)	21 (84.0)	9 (90.0)	61 (88.4)
Platelet count decreased	19 (27.5)	9 (36.0)	3 (30.0)	27 (39.1)
Electrocardiogram QT prolonged	2 (2.9)	1 (4.0)	0 (0.0)	3 (4.3)
White blood cell count decreased	43 (62.3)	14 (56.0)	7 (70.0)	44 (63.8)
Blood alkaline phosphatase increased	7 (10.1)	3 (12.0)	1 (10.0)	11 (15.9)
Metabolism and nutrition disorders				
Decreased appetite	44 (63.8)	9 (36.0)	4 (40.0)	46 (66.7)
Musculoskeletal and connective tissue disorders				
Arthralgia	1 (1.4)	2 (8.0)	2 (20.0)	5 (7.2)
Musculoskeletal pain	0 (0.0)	2 (8.0)	2 (20.0)	3 (4.3)
Myalgia	11 (15.9)	3 (12.0)	2 (20.0)	17 (24.6)
Rhabdomyolysis	1 (1.4)	0 (0.0)	0 (0.0)	1 (1.4)
Nervous system disorders				
Dysgeusia	9 (13.0)	4 (16.0)	3 (30.0)	10 (14.5)
Headache	8 (11.6)	7 (28.0)	4 (40.0)	14 (20.3)
Psychiatric disorders				
Insomnia	7 (10.1)	4 (16.0)	0 (0.0)	11 (15.9)
Skin and subcutaneous tissue disorders				
Eczema	1 (1.4)	3 (12.0)	2 (20.0)	3 (4.3)
Pruritus	1 (1.4)	1 (4.0)	2 (20.0)	4 (5.8)

**Table 3:** Treatment Cycle Delays and Dose Reductions by each Duration

	<3 mo	6‐12 mo	>12 mo	All
	(N = 69)	(N = 25)	(N = 10) ^＊^	(N = 69)
Patients with cycle delay, n (%)	50 (72.5)	22 (88.0)	7 (70.0)	51 (73.9)
Number of cycle delays, n (%)				
0	19 (27.5)	3 (12.0)	3 (30.0)	18 (26.1)
1	13 (18.8)	7 (28.0)	0 (0.0)	13 (18.8)
2	17 (24.6)	4 (16.0)	2 (20.0)	6 (8.7)
3	20 (29.0)	5 (20.0)	0 (0.0)	5 (7.2)
4	0 (0.0)	2 (8.0)	0 (0.0)	5 (7.2)
≧5	—	4 (16.0)	5 (50.0)	22 (31.9)
Patients with dose reduction, n (%)	10 (14.5)	7 (28.0)	3 (30.0)	13 (18.8)
Number of dose reduction level, n (%)				
0	59 (85.5)	18 (72.0)	7 (70.0)	56 (81.2)
−1	10 (14.5)	6 (24.0)	2 (20.0)	12 (17.4)
−2	0 (0.0)	1 (4.0)	1 (10.0)	1 (1.4)

## 4 DISCUSSION

Here, we presented the pattern of size change in STS treated by trabectedin. The detailed analysis with a long‐term follow‐up revealed that tumors shrank slowly and stably, and the response occurred in a delayed fashion from the initiation of treatment. With a thorough literature review, a patient with uterine leiomyosarcoma was reported to show marked tumor shrinkage following repeated trabectedin administration, although the tumor size remained unchanged during the early stage of therapy.^8^

Le Cesne et al reported that when trabectedin was administered as second‐ or third‐line treatment for STS, the median period from treatment to response was 5.3 months,^9^ which is close to our results. Additionally, we presented the changes in tumor diameter over time and demonstrated that the antitumor effect of trabectedin could appear after an initial trend of tumor growth. Furthermore, we encountered a unique case of myxoid liposarcoma wherein trabectedin treatment could be continued for over 1 year after which the case was rated as PD on the RECIST, although this case was ultimately not rated as a responder (Figure 5B). These findings suggest that even when tumors show a tendency for growth during the early stage of treatment, tumor shrinkage or long‐term stable disease may be achieved by continuous trabectedin treatment.

However, we could not rule out the possibility that such a delayed response is a feature of STS itself. The histological response to chemotherapy is occasionally inconsistent with tumor shrinkage as evaluated by diagnostic imaging.^10^ In Figure 3B, we showed the difference in the rate of change in tumor size between the major histological subtypes included in this study. It indicated that tumor increase before shrinkage and the delayed response were most apparent in myxoid liposarcoma. Reasons for such inconsistency could include increased volume of fluid material produced by tumor necrosis and large differences in histological features among different histological STS types (ie differences in cellularity, the ratio of tumor cells to stroma, and nature of the stroma among the histological types). Even after tumor cells lose viability due to chemotherapy, the fibrous stroma may persist, whereas the myxoid stroma may be absorbed. For example, in cases of gastrointestinal stromal tumors treated with chemotherapy, tumor density decreased owing to necrosis even in the absence of changes in diameter, resulting in patients being rated as “responders”.^11^ Thus, clinicians must understand the response pattern of each histological type of STS to each drug.

In our study, PR was observed earlier in synovial sarcomas, followed by myxoid liposarcoma and mesenchymal chondrosarcoma. Synovial sarcomas show varying cell densities and a wide range of the ratio of tumor cells to fibrous stroma, while myxoid liposarcomas have myxoid stroma with varying degrees of cellularity. Mesenchymal chondrosarcomas have a bimorphic pattern that is composed of small round cells and islands of well‐differentiated hyaline cartilage. Thus, each histological type shows unique microscopic features, possibly resulting in differences in the timing of tumor shrinkage caused by trabectedin.

The unique molecular function of trabectedin potentially influences delayed response to the treatment besides tumor histology. Trabectedin is known to show antitumor activity by inhibiting tumor cell DNA repair and by inducing apoptosis.^12^ Besides it, Galmarini et al suggested that the effect of trabectedin on the tumor microenvironment was another mechanism leading to tumor size diminishment.^13^ Altogether, these findings suggest that the delayed response to trabectedin treatment is attributed to the secondary antitumor activity of this drug on the tumor microenvironment, besides the primary antitumor effect.

We also analyzed the relation between the initial response to trabectedin and the treatment continuity. The similar method of investigating patient prognoses based on the patients’ initial response to treatment was also adopted by the European Organization for Research and Treatment of Cancer.^14^ Their study revealed that absence of progression (less than 10% increase or decrease in size) after the initial two cycles of doxorubicin‐based chemotherapy was associated with improved survival of patients with metastatic sarcoma. Our findings are relevant to their results because patients without progression (less than 20% increase or decrease in size) at week 8 show a longer stable disease than another and can continue trabectedin treatment over a prolonged period. Based on these results, absence of progression is more meaningful than how much the tumor decreases in size. It is important for clinicians to understand the time‐lapse change in size specific to each drug and manage drug administration accordingly. Even when tumors show a tendency of less than 20% growth after a few treatment cycles, trabectedin may exhibit more efficacy if used continuously, rather than immediately switching to other drugs, in cases with stable clinical symptoms.

This is the first report on the long‐term safety of trabectedin at a dose of 1.2 mg/m^2^ in Japanese patients. Unlike doxorubicin, which poses an increased cardiotoxicity risk when used over a long period, trabectedin showed no major issues associated with its long‐term use. In other words, our findings showed that trabectedin can safely be administered over a long period as long as tumors remain controlled. However, side effects, including rhabdomyolysis and elevated CPK levels, are uncommon among patients taking other anticancer agents; thus, CPK levels should regularly be monitored in patients taking trabectedin. Several patients experienced cycle delays during the early stage of treatment, which was more common among long‐term trabectedin users. Conversely, the dose reduction rate was low among long‐term trabectedin users. These findings suggest that a key factor for effective trabectedin administration is considering cycle delays for patients with a slow recovery from toxicity.

This study has some limitations. Sample size (n = 66) was not large enough for a statistical analysis, even though detailed time‐lapse analysis was able to be performed. We could not collect and compare the data on the pattern of tumor size change following treatment with the other existing drugs used for STSs. Hence, owing to lack of controls for comparison, we cannot conclude whether the delayed and persistent response is a unique finding of trabectedin. Furthermore, we have the data on translocation‐related sarcomas only. We cannot rule out the possibility that histological subtype have affected the response pattern to trabectedin. Although there are some limitations, we believe that this paper would be a valuable comparison data for the future research because there are little publicly‐available detailed data on time lapse change of tumor response in soft tissue sarcoma at this time.

Because STS contains diverse histological types, it is essential to select drug therapy based on the nature of tumor cells and the mechanism of action of the drug. Despite having a limited sample size, this analysis revealed characteristic responses of STS to trabectedin treatment. Our analysis indicated that a mild increase in tumor size alone would not be a sufficient reason to discontinue trabectedin treatment. To ensure maximum drug efficacy, we recommend accumulating experience with this therapy through clinical practice and identifying predictive factors for delayed response.

## CONFLICT OF INTEREST

Makoto Endo received grant from Japan Orthopaedics and Traumatology Research Foundation Inc; personal fee from Taisho Toyama Pharmaceutical Co., Ltd., Daiichi Sankyo Company, Limited, Eli Lilly and Company, Eisai Co., Ltd., and Novartis International AG. Shunji Takahashi received grant, personal fee from Eisai, Novartis, Daiichi‐Sankyo, Taiho, MSD, Chugai, Bayer, and Astrazeneca. Nobuhito Araki and Hideshi Sugiura have no conflicts of interest. Takafumi Ueda received grant from Eisai, Takara Bio, Eli Lilly; personal fee from Daiichi‐Sankyo and Taiho Pharmaceuticals. Tsukasa Yonemoto, Mitsuru Takahashi, and Hideo Morioka have no conflicts of interest. Hiroaki Hiraga received grant from Taiho and Eli Lilly; personal fee from Daiichi Sankyo, Ono, Eisai, Nippon Kayaku, Kaken, and Novartis. Toru Hiruma, Toshiyuki Kunisada, and Akihiko Matsumine have no conflicts of interest. Kazato Goda is employed by Taiho Pharmaceutical Co., Ltd and received personal fees from Taiho Pharmaceutical Co., Ltd both during the study period and outside the submitted work. Akira Kawai received personal fee from Taiho Pharmaceutical Co.

## AUTHOR CONTRIBUTIONS

Makoto Endo: Study concept, study design, data collection, study oversight, data analysis, writing of the original draft, and approval of the final draft. Shunji Takahashi: Provision of patients, data collection, review and editing of the initial draft, and approval of the final draft. Nobuhito Araki: Provision of patients, data collection, review and editing of the initial draft, and approval of the final draft. Hideshi Sugiura: Provision of patients, data collection, review and editing of the initial draft, and approval of the final draft. Takafumi Ueda: Provision of patients, data collection, review and editing of the initial draft, and approval of the final draft. Tsukasa Yonemoto: Provision of patients, data collection, review and editing of the initial draft, and approval of the final draft. Mitsuru Takahashi: Provision of patients, data collection, review and editing of the initial draft, and approval of the final draft. Hideo Morioka: Provision of patients, data collection, review and editing of the initial draft, and approval of the final draft. Hiroaki Hiraga: Provision of patients, data collection, review and editing of the initial draft, and approval of the final draft. Toru Hiruma: Provision of patients, data collection, review and editing of the initial draft, and approval of the final draft. Toshiyuki Kunisada: Provision of patients, data collection, review and editing of the initial draft, and approval of the final draft. Akihiko Matsumine: Provision of patients, data collection, review and editing of the initial draft, and approval of the final draft. Kazato Goda: Study concept, writing of the original draft, data interpretation, writing of the original draft, approval of the final draft, and overall management of manuscript preparation. Akira Kawai: Study design, provision of patients, data collection, study oversight, data analysis, review and editing of the initial draft, and approval of the final draft. All authors were investigators of the 2 clinical trials reported in this study.

## Supporting information

FigS1

## Data Availability

Data generated or analyzed during this study are on file with Taiho Pharmaceutical Co Ltd and are not publicly available. Inquiries for data access may be sent to the following email address: TOIAWASE@taiho.co.jp.
