# Supplementary figures and images for: Time lapse analysis of tumor response in patients with soft tissue sarcoma treated with trabectedin: A pooled analysis of two phase II clinical trials
Source: Cancer Med. 2020 Mar 27;9(11):3656–67. doi: 10.1002/cam4.2991 (PMC7286446; doi:10.1002/cam4.2991)

BSC (n = 29)

Trabectedin treatment (n = 29)

Change in target lesion size from baseline (%)

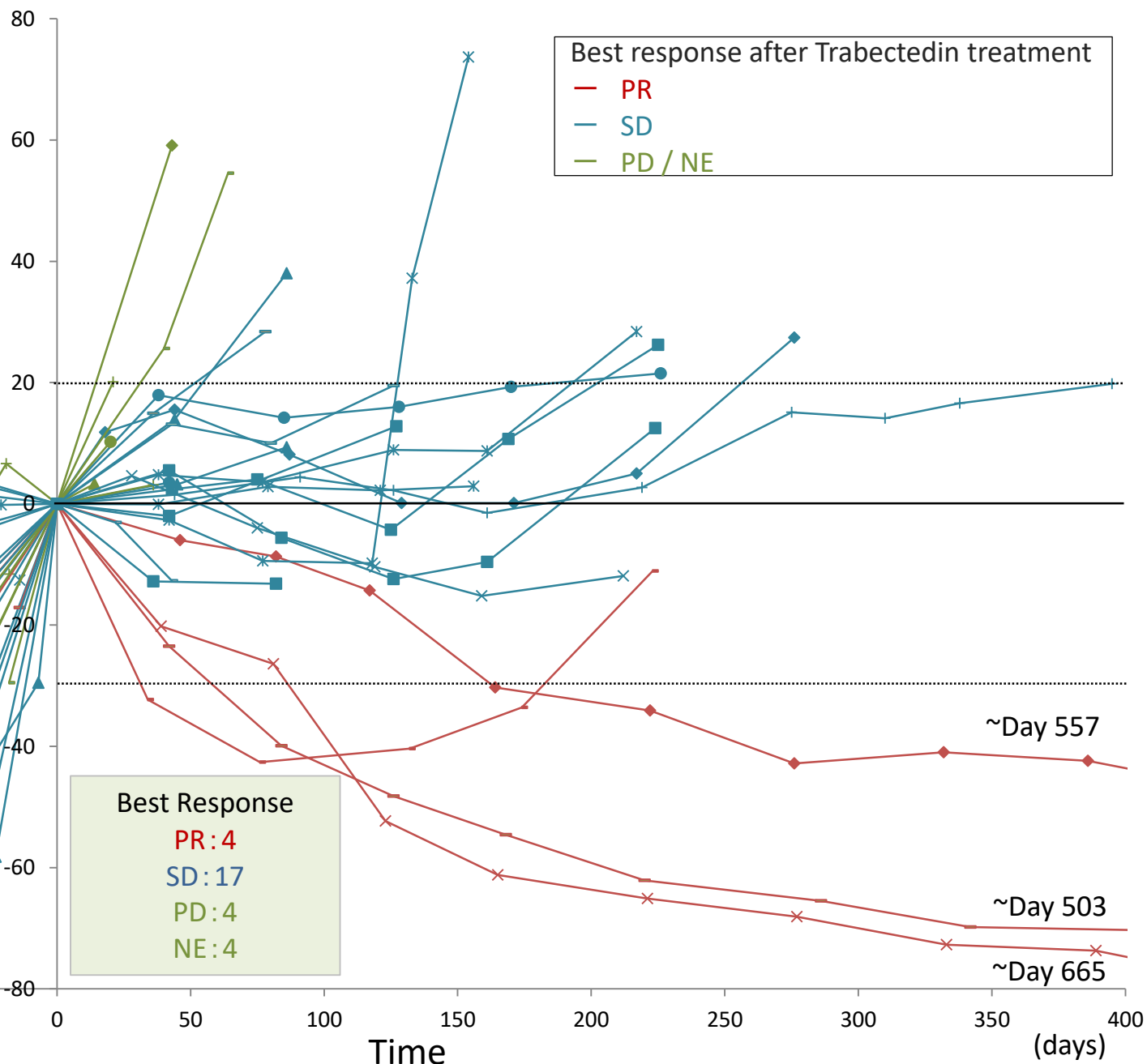

Best Response  
PD: 25  
NE: 4

Supplement: Supplementary file 1 — FigS1 [file CAM4-9-3656-s001.pdf]
